# Phosphatidylcholine suppresses inflammatory responses in LPS-stimulated MG6 microglial cells by inhibiting NF-κB/JNK/p38 MAPK signaling

**DOI:** 10.1371/journal.pone.0328206

**Published:** 2025-07-28

**Authors:** Sachiko Mizuno, Yuki Kurobe-Takashima, Daisuke Kuriki, Kenta Susaki, Kurataka Otsuka, Tomoko Tsuchihashi, Keiko Abe, Shoko Kobayashi

**Affiliations:** 1 Graduate School of Agricultural and Life Sciences, The University of Tokyo, Bunkyo-ku, Tokyo, Japan; 2 R&D Division, Kewpie Corporation, Tokyo, Japan; 3 Division of Translational Oncology, Fundamental Innovative Oncology Core, National Cancer Center Research Institute, Tokyo, Japan; 4 Food Safety Science Center, Quality Assurance Division, Kewpie Corporation, Tokyo, Japan; 5 Faculty of Fisheries Sciences, Hokkaido University, Minato, Hakodate, Japan; Universidade Federal do Rio de Janeiro, BRAZIL

## Abstract

Phosphatidylcholine (PC), a choline-containing phospholipid abundant in chicken eggs, is widely consumed as a dietary supplement. Epidemiological studies suggest that PC intake may improve cognitive function in patients with neurodegenerative diseases such as Alzheimer’s disease, although the underlying mechanisms remain largely unclear. In this study, we investigated the anti-inflammatory effects of PC and its molecular mechanisms using an in vitro inflammation model involving lipopolysaccharide (LPS)-stimulated MG6 mouse microglial cells. PC significantly suppressed the LPS-induced expression of pro-inflammatory cytokines, including tumor necrosis factor-α (TNF-α), interleukin-1β (IL-1β), and interleukin-6 (IL-6). Mechanistically, PC inhibited the phosphorylation of inhibitor kappa Bα (IκBα), thereby preventing the nuclear translocation of nuclear factor-κB (NF-κB). PC also reduced the phosphorylation of c-Jun N-terminal kinase (JNK) and p38 mitogen-activated protein kinase (MAPK), and suppressed the nuclear translocation of activator protein-1 (AP-1), composed of c-Fos and c-Jun. These findings indicate that PC attenuates LPS-induced microglial inflammation via the NF-κB and JNK/p38 MAPK signaling pathways. Given the proposed role of chronic neuroinflammation in the progression of neurodegenerative diseases, the anti-inflammatory properties of PC demonstrated here may provide new insights into its potential contribution to maintaining brain health.

## Introduction

As the global population ages, the incidence of neurodegenerative diseases such as Alzheimer’s disease (AD), Parkinson’s disease, and multiple sclerosis continues to increase [1]. These diseases are pathologically characterized by the slow and progressive loss of neuronal structure and function in the central nervous system (CNS) [2]. Although the precise causes of neurodegeneration remain unclear [3], accumulating evidence indicates that neuroinflammation plays a central role in the development and progression of neurodegenerative disorders [4].

Microglia, the resident immune cells of the CNS, are key mediators of neuroinflammation [5]. They migrate through the CNS, release cytokines, phagocytose debris and pathogens, and prune synapses. While these functions are essential for maintaining homeostasis, excessive or uncontrolled microglial activation can lead to chronic inflammation and contribute to neurodegenerative processes [4,6]. Thus, modulating microglia-mediated neuroinflammation is considered a promising strategy for preventing or slowing neurodegeneration.

In AD, one notable pathological feature is the reduction of the neurotransmitter acetylcholine in the brain, and the resulting cholinergic dysfunction is thought to underlie cognitive symptoms [7]. For this reason, dietary supplementation with choline, a precursor of acetylcholine, has long been explored as a potential therapeutic approach [8]. Phosphatidylcholine (PC), a major dietary source of choline found abundantly in chicken eggs, is widely consumed as a nutritional supplement [9,10]. Animal studies have suggested that PC supplementation may reduce neural damage and mitigate cognitive decline [11], and some epidemiological studies have reported improved cognitive performance in AD patients following PC intake [12]. However, the molecular mechanisms underlying these effects remain largely unexplored.

Lipopolysaccharide (LPS), a component of gram-negative bacterial cell walls, is commonly used to induce microglial activation and model neuroinflammatory responses in vitro [13,14]. Upon binding to toll-like receptor 4 (TLR4) on the microglial surface, LPS activates downstream signaling pathways via adaptor proteins such as myeloid differentiation factor 88 (MyD88), leading to the phosphorylation of mitogen-activated protein kinases (MAPKs) and activation of nuclear factor-κB (NF-κB) [15–17]. Phosphorylated MAPKs trigger the activation and nuclear translocation of activator protein-1 (AP-1), composed of c-Jun and c-Fos dimers, promoting the expression of proinflammatory cytokines [18]. Meanwhile, phosphorylated IκBα undergoes degradation, allowing NF-κB to translocate into the nucleus and further amplify inflammatory gene expression [19]. Therefore, suppressing the LPS-induced release of inflammatory mediators from microglia is considered a viable approach to attenuating CNS inflammation associated with diseases such as AD [17].

In this study, we investigated the anti-inflammatory effects of PCin an in vitro model of LPS-induced neuroinflammation using mouse microglial MG6 cells. We also aimed to elucidate the molecular pathways underlying PC-mediated suppression of inflammation, thereby contributing to a deeper understanding of how PC may help maintain brain health in the context of neurodegenerative conditions.

## Materials and methods

### Reagents

PC (cat#27554−14) from an egg yolk source and high-glucose Dulbecco’s Modified Eagle Medium (DMEM) were acquired from Nacalai Tesque (Kyoto, Japan). Fetal bovine serum (FBS) was obtained from Life Technologies (Carlsbad, CA, USA). LPS and insulin were purchased from Sigma-Aldrich (Tokyo, Japan), and β-mercaptoethanol was acquired from Fujifilm Wako Pure Chemicals (Osaka, Japan). Penicillin and streptomycin were purchased from Thermo Fisher Scientific (Waltham, MA, USA).

### Analysis of the fatty acid composition of PC

150 mg of L-α-phosphatidylcholine (Nakalai Tesque was added to 4 mL of sodium hydroxide (0.3125 mol/L) prepared in methanol. The mixture in an eggplant flask was heated to 80°C in a water bath for 10–15 min. After adding 5 mL of boron trifluoride methanol (Sigma-Aldrich) to the mixture and boiling for 2 min, 5 mL of hexane (Kanto Chemical, Tokyo, Japan) was added, and the mixture was boiled for 1 min. After cooling the mixture, the hexane layer was washed 3 − 4 times with ultrapure water, added to sodium sulfate (Kanto Chemical), and shaken lightly. The resulting solution was injected into an Agilent Technologies 8860 GC System (Santa Clara, CA, USA) fitted with a Supelco 24056 capillary column (100 m × 0.25 mm × 0.20 μm, Sigma-Aldrich). The composition of the fatty acid was analyzed based on the peak area obtained.

### Cell culture

The murine microglial cell line MG6 was obtained from RIKEN Cell Bank (Saitama, Japan). MG6 cells were cultured in DMEM supplemented with 10% FBS, 10 µg/mL insulin, 100 µM β-mercaptoethanol, 100 U/mL penicillin, and 0.1 mg/mL streptomycin. Cell culture was performed at 37°C under 5% CO_2_ conditions and in a humidified environment. MG6 cells were pretreated for 1 h with 50 or 100 μM PC or vehicle control, followed by stimulation with 100 ng/mL of LPS or LPS vehicle for 24 h in the continued presence of PC or PC vehicle.

### Cell viability assay

To determine the cytotoxicity of PC in MG6 microglial cells, MG6 microglial cells were seeded at a density of 1 × 10^4^ cells/well in 96-well plates and treated with various concentrations of PC (5, 20, 30, 50, and 100 μM) for 24 h. The MTT [3-(4,5-dimethylthiazol-2-yl)-2,5-diphenyltetrazolium bromide] cell viability assay kit (BIOTIUM Inc., Fremont, CA, USA) was used to measure the cells according to the manufacturer’s protocol.

### RT-qPCR analysis

MG6 cells were seeded at a density of 2.5 × 10^5^ cells/well in 24-well plates in a serum-free medium. The cells were pre-incubated alone or with PC for 1 h and then stimulated with LPS (100 ng/mL) for 24 h. Total RNA isolation from MG6 cells was performed using the RNeasy^®^ Mini Kit (QIAGEN, Hilden, Germany). Subsequently, reverse transcription was performed using the PrimeScript™ RT Master Mix (Takara, Shiga, Japan). RT-qPCR analysis was performed using a Thermal Cycler Dice^®^ Real-Time PCR system (Takara) with TB Green^™^ Premix Ex Taq^™^ II (Takara) and gene-specific primers, which are listed in Table 1. Gene expression levels were normalized to β-actin, and the relative expression was determined using the 2^-∆∆Ct^ method.

**Table 1:** Sequences of the primers used for RT-qPCR.

Gene	Forward primer (5′-3′)	Reverse primer (5′-3′)
*TNFα*	GGTGCCTATGTCTCAGCCTCTT	GCCATAGAACTGATGAGAGGGAG
*IL-1β*	TGGACCTTCCAGGATGAGGACA	GTTCATCTCGGAGCCTGTAGTG
*IL-6*	TACCACTTCACAAGTCGGAGGC	CTGCAAGTGCATCATCGTTGTTC
*β-actin*	GGCTGTATTCCCCTCCATCG	CCAGTTGGTAACAATGCCATGT

### Western blot analysis

MG6 cells were cultured in a 100-mm dish at a density of 6.0 × 10^6^ cells/dish in serum-free medium, pre-incubated alone or with PC for 24 h, and then stimulated with LPS (100 ng/mL) for 1 h. Subsequently, whole cell proteins were extracted using RIPA buffer (Fujifilm Wako Pure Chemicals), and then the cytoplasmic and nuclear proteins were extracted using the NE-PER Nuclear and Cytoplasmic Extraction Reagent Kit (Thermo Fisher Scientific), according to the manufacturer’s instructions. The protein concentration was determined using the Pierce™ BCA Protein Assay Kit (Thermo Fisher Scientific). Samples were subjected to SDS-PAGE separation, followed by transfer onto iBlot™ 3 Transfer Stacks Midi PVDF membranes using the iBlot™ Western Blot Transfer System (Thermo Fisher Scientific). For blocking, the membrane was treated with Bullet Blocking One for Western Blotting (Nacalai Tesque) for 5 min at room temperature. After overnight incubation at 4°C with the primary antibody, the membranes were incubated with horseradish peroxidase (HRP)-conjugated anti-rabbit (cat# 65–6120, Invitrogen, Carlsbad, CA, USA) or anti-mouse (cat# 31430, Invitrogen) secondary antibody at a dilution of 1:5000 at room temperature for 1 h. Details of the primary antibodies and their respective working dilutions are listed in Table 2. Protein bands were visualized using the ImageQuant™ LAS 4000 system (Fujifilm, Tokyo, Japan) with ECL™ Prime Western Blotting Detection Reagents (Cytiva, Tokyo, Japan). Image adjustments were applied uniformly across the entire blot using ImageJ software (version 1.54).

**Table 2:** Primary antibody.

Name	Company	Concentration	Product number
TNFα	Proteintech	1:1000	60291-1-Ig
IL-1β	Cell Signaling	1:500	12242
IL-6	Santa Cruz Biotechnology	1:1000	sc-57315
NF-κB p65	Cell Signaling	1:1000	8242
p-IκBα	Cell Signaling	1:2000	9246
IκBα	Cell Signaling	1:2000	4814
p-p38 MAPK	Santa Cruz Biotechnology	1:500	sc-166182
p38 MAPK	Santa Cruz Biotechnology	1:2000	sc-271120
p-JNK	Cell Signaling	1:500	9251
JNK	Cell Signaling	1:1000	9252
β-actin	Cell Signaling	1:5000	4970
TLR4	Santa Cruz Biotechnology	1:2000	sc-293072
MyD88	Cell Signaling	1:1000	4283
c-Fos	Cell Signaling	1:1000	2250
c-Jun	Cell Signaling	1:2000	9165
Lamin B1	Santa Cruz Biotechnology	1:200	sc-374015

Western blotting was performed using protein samples prepared from two independent experimental batches. Equal amounts of protein (10 µg per lane) were loaded onto SDS-PAGE gels and transferred to PVDF membranes under identical electrophoresis and transfer conditions within each batch. Due to the large number of target proteins and the similarity in their molecular weights, separate membranes were used for the detection of each target protein in a given batch. This also applied to loading controls (e.g., β-actin for both batches A and B, and Lamin B1 for batch B), which were each detected on their own dedicated membranes. All membranes within the same batch were processed in parallel using identical blocking, antibody incubation, washing, and detection protocols. Specifically, Fig 2 shows results from batch A, while Figs 3–6 are derived from batch B. Western blot images for all proteins are provided in the supplementary data (S1 Raw Images and S2 Raw Data).

**Fig 1:**
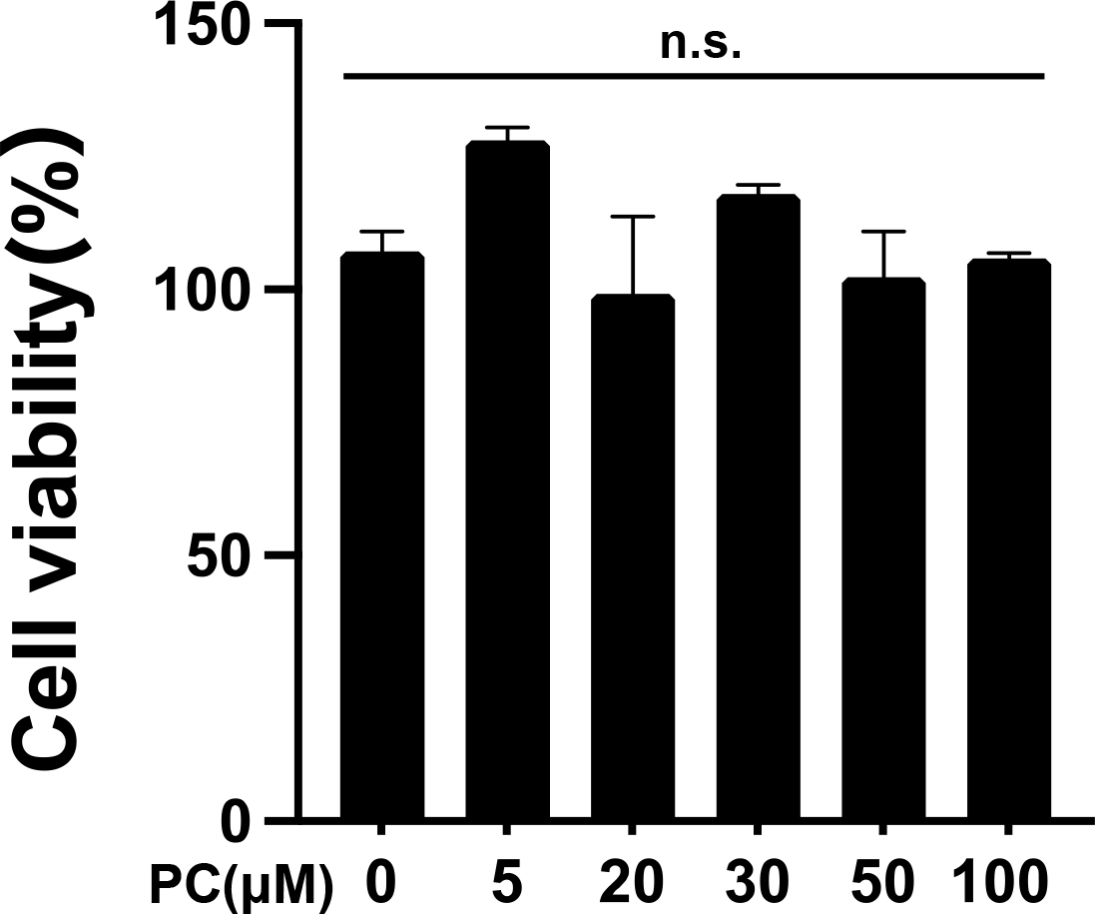
Effect of phosphatidylcholine (PC) on the cell viability of MG6 microglial cells. The MTT assay was used to measure the viability of MG6 cells. MG6 cells were treated with PC at the indicated doses (0, 5, 20, 30, 50, and 100 μM) for 24 h. Quantitative data are expressed as a percentage corresponding to the raw control value. Data were analyzed using a one-way ANOVA followed by Dunnett’s tests and are presented as the mean ± standard error of the mean (SEM) from three independent experiments. n.s., no significance. The raw data necessary to replicate the findings are available in S2 Raw Data.

**Fig 2:**
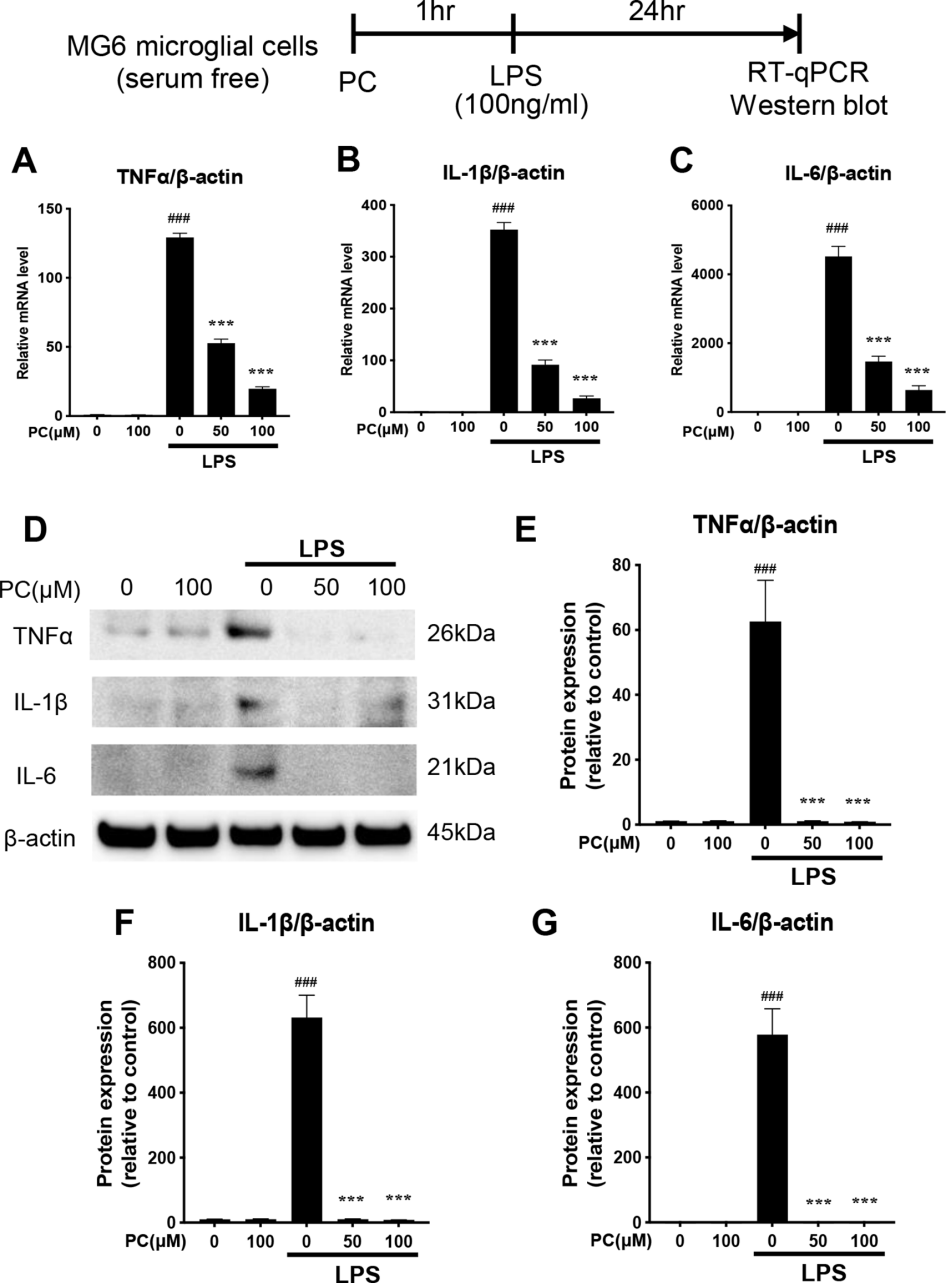
Phosphatidylcholine (PC) reduced the lipopolysaccharide (LPS)-induced expression of proinflammatory cytokines in MG6 cells. MG6 microglial cells were incubated for 24 h with LPS (100 ng/mL) alone or after 1 h of pretreatment with different amounts of PC. The mRNA and whole-cell proteins were then extracted. The mRNA expression levels of *TNF-α* (A), *IL-1β* (B), and *IL-6* (C) were measured by RT-qPCR, and *β-actin* was used as the internal control for analysis. The protein expression levels were measured by western blot analysis (D). The band intensities of TNF-α (E), IL-1β (F), and IL-6 (G) were quantified using Image J software, and β-actin was used as the loading control. Equal amounts of total protein (10 µg per lane), prepared from the same experimental batch (A), were subjected to SDS-PAGE and transferred to PVDF membranes. Due to the similar molecular weights of the target proteins, including the loading control β-actin, each protein was detected on a separate membrane. All membranes were processed in parallel under identical conditions for blocking, antibody incubation, washing, and signal detection. Molecular weight markers are indicated on the right. Data were analyzed using a one-way ANOVA followed by Dunnett’s tests and are presented as the mean ± standard error of the mean (SEM) from three independent experiments. ###p < 0.001 and ***p < 0.001 vs. control and LPS-treated cells, respectively. The data required to reproduce the results of this study are provided in S2 Raw Data, and the uncropped Western blot images are available in S1 Raw Images.

**Fig 3:**
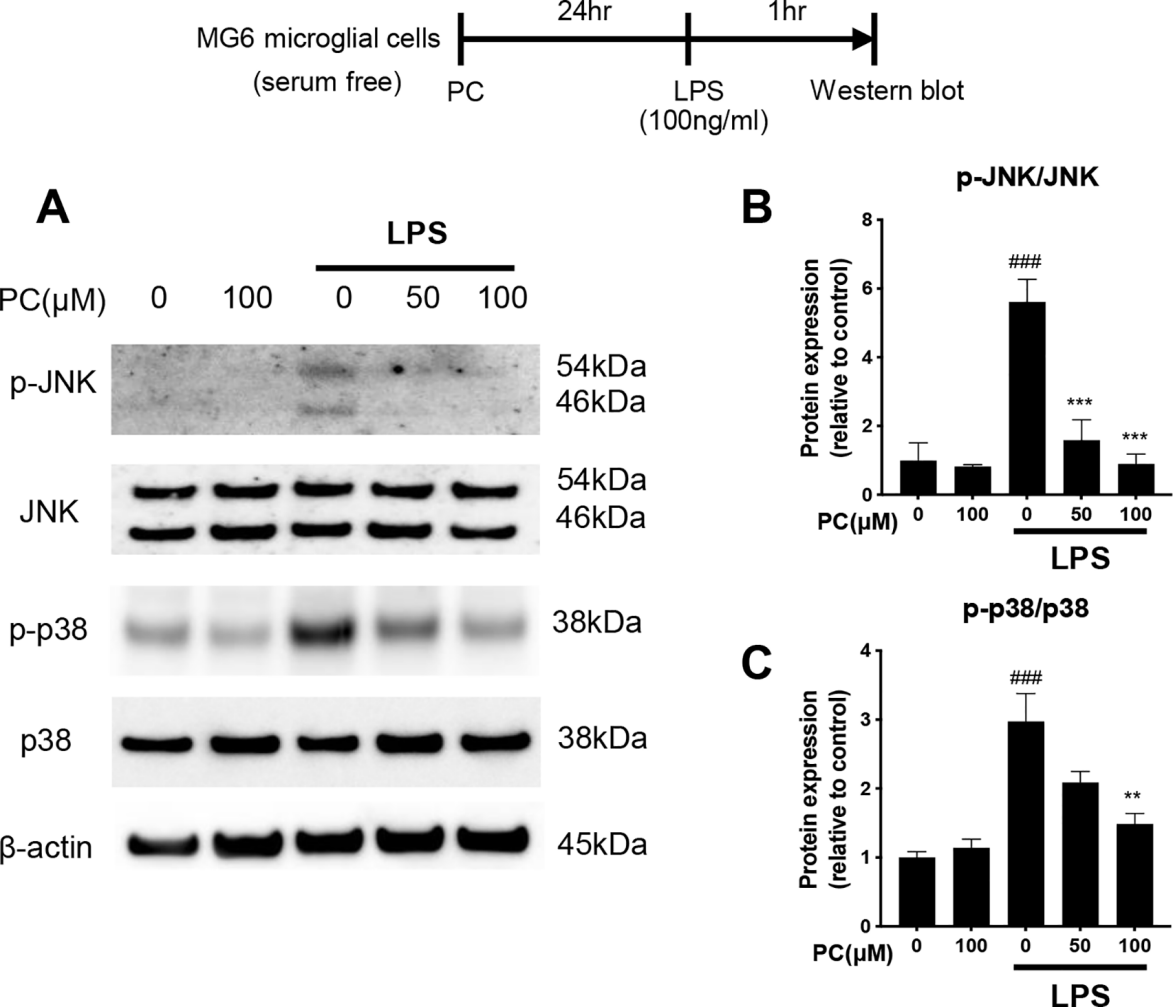
Phosphatidylcholine (PC) reduced the lipopolysaccharide (LPS)-induced JNK/p38 MAPK pathway activation in MG6 cells. MG6 cells were pre-incubated alone or with PC for 24 h and then stimulated with LPS (100 ng/mL) for 1 h. Whole-cell proteins were extracted and subjected to western blot analysis (A). The band intensities of p-JNK/JNK (B) and p-p38/p38 (C) were quantified using Image J software, and β-actin was used as the loading control. Equal amounts of total protein (10 µg per lane), prepared from the same batch experiment (B), were subjected to SDS-PAGE and transferred to PVDF membranes. The target protein was detected on a membrane processed independently under identical conditions for blocking, antibody incubation, washing, and signal detection. β-actin, used as a loading control, was detected on a separate membrane processed in parallel. The same β-actin blot is also shown in Figs 4 and 6, as all samples were derived from the same experimental batch and processed simultaneously. Data were analyzed using a one-way ANOVA followed by Dunnett’s tests and are presented as the mean ± standard error of the mean (SEM) from three independent experiments. ###p < 0.001, ***p < 0.001, and **p < 0.01 vs. control and LPS-treated cells, respectively. The data required to reproduce the results of this study are provided in S2 Raw Data, and the uncropped Western blot images are available in S1 Raw Images.

**Fig 4:**
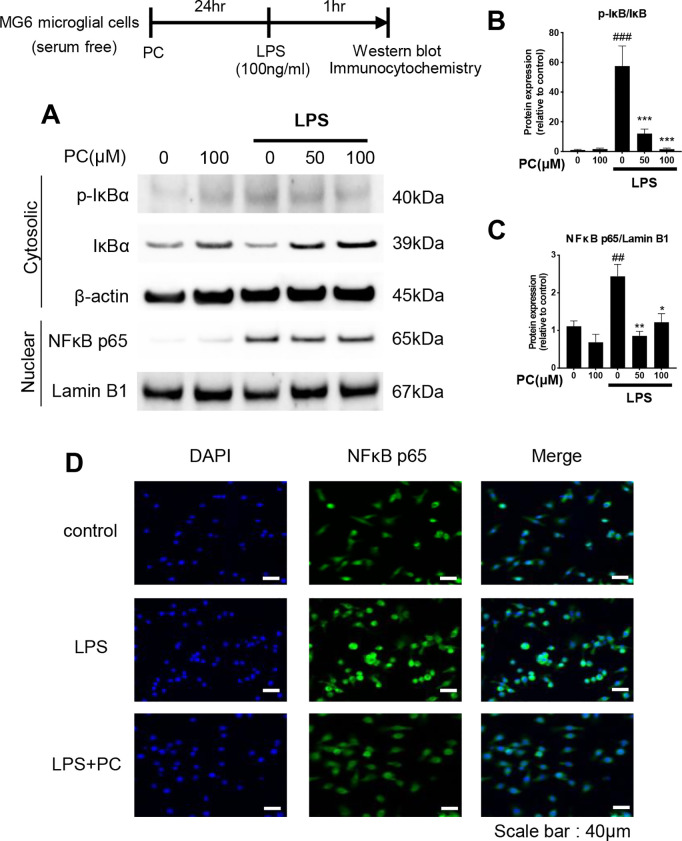
Inhibition of NF‑κB nuclear translocation by phosphatidylcholine (PC) in lipopolysaccharide (LPS)‑stimulated MG6 cells. MG6 cells were pre-incubated alone or with PC for 24 h and then stimulated with LPS (100 ng/mL) for 1 h. Nuclear and cytoplasmic proteins were isolated and subjected to western blot analysis (A). The band intensities of p-IκBα/IκBα (B) and NF-κB p65/Lamin B1 (C) were quantified using Image J software, and β-actin and Lamin B1 were used as the loading controls for the cytoplasmic and nuclear fractions, respectively. Equal amounts of total protein (10 µg per lane), prepared from the same batch experiment (B), were subjected to SDS-PAGE and transferred to PVDF membranes. The target protein was detected on a membrane processed independently under identical conditions. β-actin and Lamin B1 were used as loading controls and detected on separate membranes processed in parallel. The same β-actin blot is also shown in Figs 3 and 6, and the same Lamin B1 blot is used in Fig 5, as all data were obtained from the same experimental batch and processed simultaneously. Data were analyzed using a one-way ANOVA followed by Dunnett’s tests and are presented as the mean ± standard error of the mean (SEM) from three independent experiments. ###p < 0.001, ##p < 0.01, ***p < 0.001, **p < 0.01, and *p < 0.05 vs. control and LPS-treated cells, respectively. The localization of NF-κB p65 protein in MG6 cells was immunofluorescently stained, and the nuclei were stained with DAPI (D). The cells were then imaged by fluorescence microscopy. Scale bar = 40 µm. The data required to reproduce the results of this study are provided in S2 Raw Data, and the uncropped Western blot images are available in S1 Raw Images.

**Fig 5:**
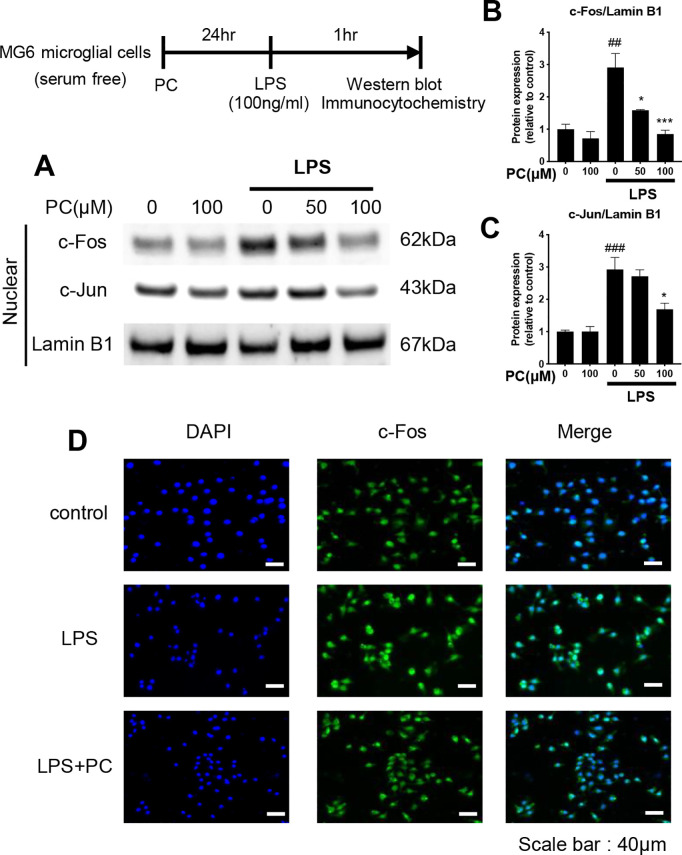
Phosphatidylcholine (PC) decreases the nuclear c-Fos and c-Jun protein levels under lipopolysaccharide (LPS) stimulation in MG6 cells. MG6 cells were pre-incubated alone or with PC for 24 h and then stimulated with LPS (100 ng/mL) for 1 h. Nuclear and cytoplasmic proteins were isolated and subjected to western blot analysis (A). The band intensities of c-Fos/Lamin B1 (B) and c-Jun/Lamin B1 (C) were quantified using Image J software, and Lamin B1 was used as the loading control. Equal amounts of total protein (10 µg per lane), prepared from the same batch experiment (B), were subjected to SDS-PAGE and transferred to PVDF membranes. The target protein was detected on a membrane processed independently under identical conditions. Lamin B1, used as a loading control, was detected on a separate membrane processed in parallel. The same Lamin B1 blot is also shown in Fig 4, as all samples were derived from the same experimental batch and processed simultaneously. Data were analyzed using a one-way ANOVA followed by Dunnett’s tests and are presented as the mean ± standard error of the mean (SEM) from three independent experiments. ###p < 0.001, ##p < 0.01, ***p < 0.001, and *p < 0.05 vs. control and LPS-treated cells, respectively. The localization of c-Fos protein in MG6 cells was immunofluorescently stained, and the nuclei were stained with DAPI (D). The cells were then imaged with fluorescence microscopy. Scale bar = 40 µm. The data required to reproduce the results of this study are provided in S2 Raw Data, and the uncropped Western blot images are available in S1 Raw Images.

**Fig 6:**
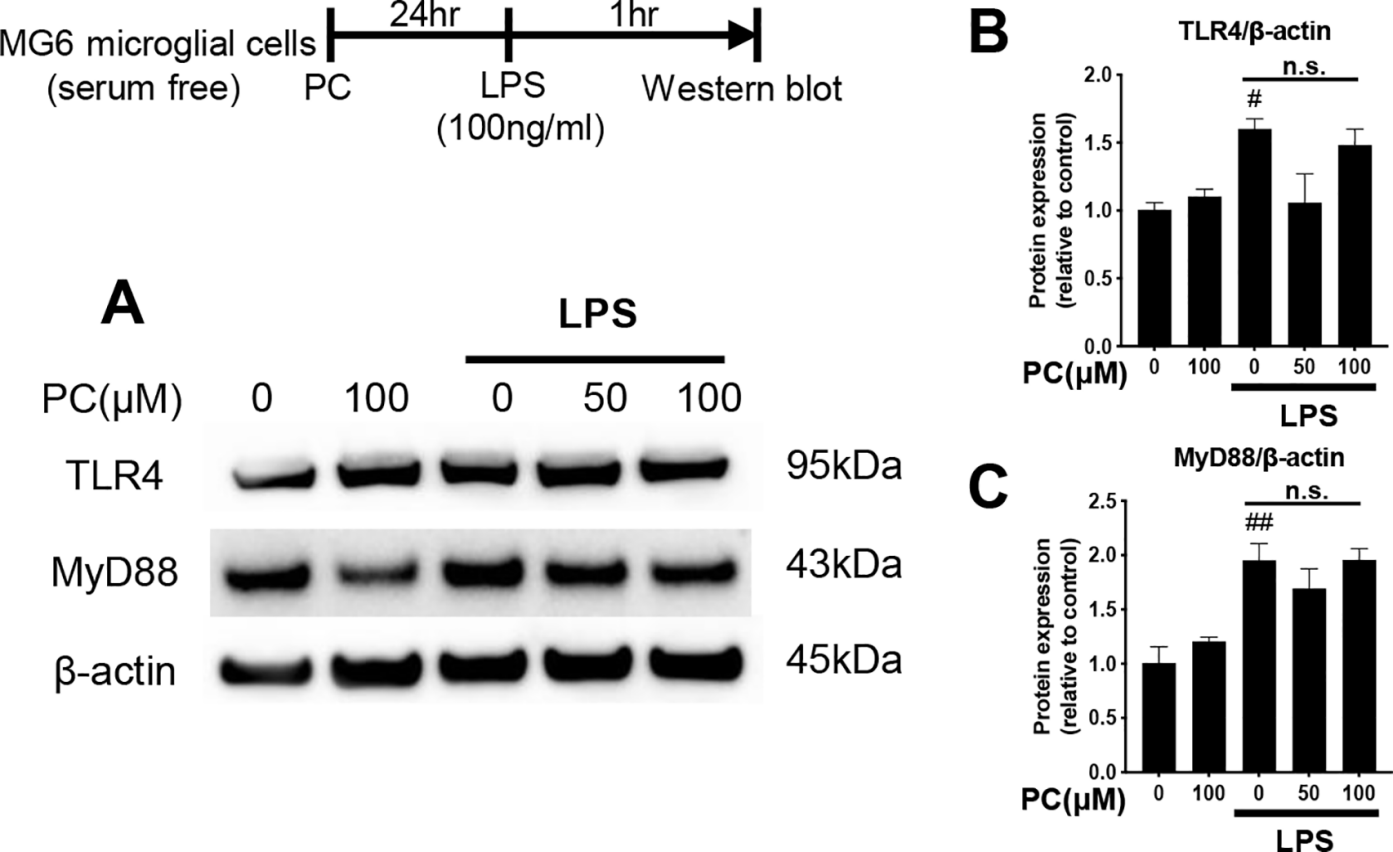
Phosphatidylcholine (PC) does not affect TLR4 and MyD88 protein expression in lipopolysaccharide (LPS)-stimulated MG6 cells. MG6 cells were pre-incubated alone or with PC for 24 h and then stimulated with LPS (100 ng/mL) for 1 h. Whole-cell proteins were isolated and then subjected to western blot analysis (A). The band intensities of TLR4/β-actin (B) and MyD88/β-actin (C) were quantified using Image J software, and β-actin was used as the loading control. Equal amounts of total protein (10 µg per lane), prepared from the same batch experiment (B), were subjected to SDS-PAGE and transferred to PVDF membranes. The target protein was detected on a membrane processed independently under identical conditions. β-actin, used as a loading control, was detected on a separate membrane processed in parallel. The same β-actin blot is also shown in Figs 3 and 4, as all samples were derived from the same experimental batch and processed simultaneously. Data were analyzed using a one-way ANOVA followed by Dunnett’s tests and are presented as the mean ± standard error of the mean (SEM) from three independent experiments. ##p < 0.01 and #p < 0.05 vs. control. n.s., no significance (S1 Fig). The data required to reproduce the results of this study are provided in S2 Raw Data, and the uncropped Western blot images are available in S1 Raw Images.

### Immunofluorescence staining

MG6 cells were cultured at a density of 2.5 × 10^5^ cells/well on a 4-well Slide & Chamber (WATSON CO., LTD, Tokyo, Japan) in serum-free medium, preincubated alone or with PC for 24 h and then stimulated with LPS (100 ng/mL) for 1 h. Subsequently, the cells were fixed with 4% paraformaldehyde for 15 min, washed three times with phosphate-buffered saline (PBS), permeabilized with 0.1% Triton X-100 (Sigma-Aldrich) for 5 min, and incubated with EzBlock BSA (ATTO, Tokyo, Japan) for 1 h at room temperature. The cells were incubated overnight with NF-κB p65 antibody (1:250 dilution) or c-Fos antibody (1:250 dilution) in the blocking solution at 4°C. After washing three times with PBS, the cells were incubated with multi-rAb CoraLite Plus 488-goat antimouse (cat# RGAM002, Proteintech, Rosemont, IL, USA) or Alexa Fluor® 488-conjugated goat anti-rabbit IgG (cat# ab150077, abcam, Cambridge, UK) secondary antibodies at a dilution of 1:400 and counterstained with DAPI (Thermo Fisher Scientific) in the dark at room temperature for 1 h. The coverslips were mounted with Fluoromount^™^ Aqueous Mounting Medium (Sigma-Aldrich), and fluorescence images were visualized using a microscope (OLYMPUS, Tokyo, Japan).

### Statistical analysis

The data are presented as the mean ± standard error of the mean (SEM) derived from at least three independent replicates. All data were analyzed with a one-way ANOVA followed by Dunnett’s tests. All data were analyzed using Graph Pad Prism Ver. 10.4.0 (Graph Pad Software, Inc., San Diego, CA). A p-value < 0.05 was considered statistically significant.

## Results

### Fatty acid composition of PC

The fatty acid composition of the PC used in this study was analyzed using gas chromatography-mass spectrometry (Table 3). The ratio of saturated to unsaturated fatty acids in the sample was 50:50. Palmitic acid (41.941% ± 0.237%) was the predominant saturated fatty acid present, while oleic acid (29.490% ± 0.118%) was the most abundant unsaturated fatty acid. Other unsaturated fatty acids included linoleic acid (8.751% ± 0.034%), arachidonic acid (1.655% ± 0.014%), and docosahexaenoic acid (0.318 ± 0.002%).

### Effects of PC on the viability of MG6 cells

To evaluate the potential cytotoxicity of PC in MG6 microglial cells, MG6 cells were treated with different doses of PC (5, 20, 30, 50, and 100 μM) for 24 h. Cell viability was measured using the MTT assay kit. The results showed that PC was not cytotoxic to MG6 microglial cells in the MTT assay at doses of 5–100 μM (Fig 1).

### Effects of PC on the LPS-stimulated production of proinflammatory mediators in MG6 cells

The effect of PC on LPS-induced inflammatory mediator production was examined by RT-qPCR and western blot analysis. LPS stimulation greatly increased the mRNA expression of *tumor necrosis factor (TNF)-α*, *interleukin (IL)-1β*, and *IL-6* in MG6 cells, while PC treatment decreased TNF-α, IL-1β, and IL-6 in a dose-dependent manner (Fig 2A–2C). Next, in MG6 cells, LPS stimulation increased the total cellular protein expression of TNF-α, IL-1β, and IL-6, which was dramatically and significantly suppressed by the presence of PC (50 and 100 μM) (Fig 2D–2G). These results indicate that PC treatment suppressed the expression of TNF-α, IL-1β, and IL-6 in LPS-stimulated microglial cells, which indicates its anti-inflammatory effect.

### PC inhibits the LPS-induced activation of JNK and p38 MAPK

The MAPK signaling pathway is a key pathway that is responsible for the regulation of inflammatory cytokines. MAPK, including three major subfamilies of p38 mitogen-activated protein kinase (p38 MAPK), c-Jun N-terminal kinase (JNK), and extracellular signal-regulated protein kinase (ERK), are known to be involved in inflammation-induced cascades. Previous studies have reported that neuronal cell death caused by activated microglia is due to p38 MAPK and JNK [20,21]. Therefore, we assessed whether the MAPK pathway is associated with the anti-inflammatory activity of PC by western blot analysis in MG6 cells in terms of JNK and p38 MAPK phosphorylation. Stimulation of MG6 cells with LPS increased the whole-cell protein expression of phosphorylated JNK and phosphorylated p38 MAPK (Fig 3A–3C). PC inhibited the phosphorylation of JNK by LPS in a dose-dependent manner (Fig 3B), and treatment with 100 μM PC also inhibited the phosphorylation of p38 MAPK (Fig 3C). These results indicate that regulation of JNK and p38 MAPK signaling cascades is a possible mechanism of PC’s inhibitory effect in LPS-stimulated microglia.

### PC alleviates LPS‑induced NF‑κB nuclear translocation

In microglia activated by LPS stimulation, IκBα bound to NF-κB in the cytoplasm is degraded by phosphorylation, and free NF-κB is translocated to the nucleus, thereby causing the activation of proinflammatory cytokines [19]. To investigate whether PC has an effect on IκBα phosphorylation and the nuclear translocation of NF-κB in LPS-activated MG6 cells, we performed western blot analysis on cytoplasmic protein expression of phosphorylated IκBα and the nuclear protein expression of NF-κB p65, a major subunit of NF-κB in inflammatory responses. Stimulation of MG6 cells with LPS resulted in the degradation of cytoplasmic IκBα and increased the protein expression of phosphorylated IκBα and nuclear NF-κB p65 (Fig 4A–4C). PC inhibited the phosphorylation of cytoplasmic IκBα by LPS in a dose-dependent manner (Fig 4B). PC (50 and 100 μM) also inhibited the levels of nuclear NF-κB p65 (Fig 4C). To further confirm these findings, we performed immunofluorescent staining for NF-κB p65 localization. The fluorescence intensity of the nucleus that overlapped with NF-κB p65 was increased in the LPS-stimulated cells (Fig 4D, LPS Merge) but was suppressed by treatment with 100 μM PC (Fig 4D, LPS + PC Merge). These results indicate that PC inhibits the nuclear translocation of NF-κB by impeding IκBα phosphorylation.

### PC decreases the c-Fos and c-Jun nuclear protein levels under LPS stimulation

AP-1 is downstream of MAPK and is a central transcription factor that activates inflammatory cytokines [22]. AP-1 is composed of c-Jun and c-Fos dimers and is present as a quiescent form in the cytoplasm of unstimulated cells, but upon stimulation and subsequent phosphorylation, it translocates to the nucleus and promotes the generation of inflammation-inducing mediators [23]. Therefore, the effects of PC on the translocation of c-Fos and c-Jun protein to the nucleus in LPS-stimulated MG6 cells were evaluated by western blot analysis. LPS stimulation increased the nuclear protein expression of both c-Fos and c-Jun in MG6 microglial cells (Fig 5A–5C). However, PC treatment dramatically suppressed the nuclear protein level of c-Fos in a dose-dependent manner (Fig 5B), while the nuclear protein level of c-Jun was suppressed by treatment with 100 μM PC (Fig 5C). The spatial distribution of c-Fos was confirmed by immunofluorescent staining. LPS stimulation increased the translocation of c-Fos to the nucleus in MG6 cells because the signals of the nucleus and c-Fos overlapped (Fig 5D, LPS Merge), but treatment with 100 μM PC inhibited this overlap (Fig 5D, LPS + PC Merge). These results indicate that PC inhibits the nuclear translocation of c-Fos and c-Jun.

### Effects of PC on the LPS-induced activation of TLR4 and MyD88

Previous studies have shown that the LPS-stimulated inflammatory response triggers downstream signaling via TLR4 and its adaptor protein, MyD88, on the surface of microglia, which leads to the activation of inflammatory cytokines [24]. Therefore, we evaluated whether PC treatment affected TLR4 and MyD88 in LPS-stimulated MG6 cells using western blot analysis. LPS stimulation increased the whole-cell protein expressions of TLR4 and MyD88 (Fig 6A–6C). Pretreatment with PC (50 and 100 μM) did not affect the protein expression levels of TLR4 and MyD88. These results suggest that PC may exhibit anti-inflammatory activity by affecting the downstream signaling molecules, such as IκBα, NF-κB, and MAPK, without affecting TLR4 and MyD88.

## Discussion

This study found that PC strongly suppressed the activation of IκBα and JNK/p38 MAPK, inhibited the nuclear translocation of NF-κB p65, and suppressed the formation of the AP-1 dimer (c-Fos and c-Jun), thereby reducing the release of inflammatory cytokines in LPS-stimulated MG6 microglial cells. Microglia activated by LPS stimulation led to the production of proinflammatory cytokines and mediators, such as TNF-α, IL-6, and IL-1β, and their chronic increase predisposes the CNS to neuroinflammation, which may result in the development of neurodegenerative diseases, such as AD [25,26]. PC does not exhibit cytotoxicity (Fig 1) and significantly inhibited the production of TNF-α, IL-6, and IL-1β in LPS-stimulated MG6 microglial cells (Fig 2). These results indicate that PC had an anti-inflammatory effect on LPS-activated MG6 microglial cells. To investigate the mechanism of the anti-inflammatory effect of PC on the microglial cells, we examined the effect of PC on LPS stimulation of the inflammatory intracellular signaling pathways.

**Fig 7:**
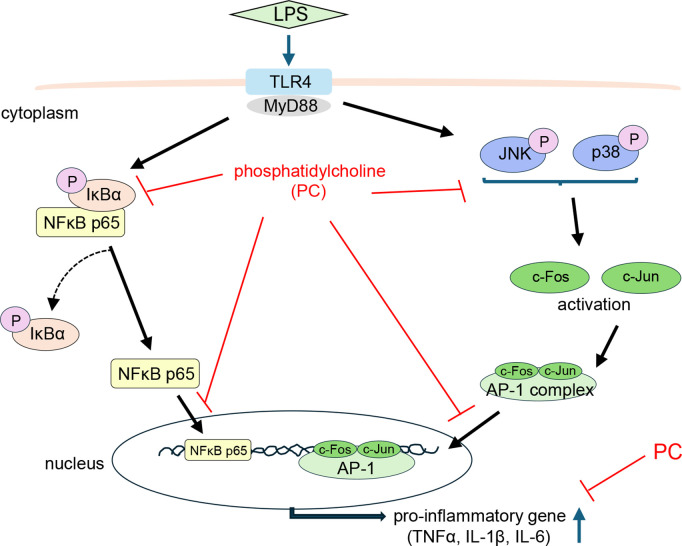
Schematic showing the cellular mechanisms involved in the anti-inflammatory effects of phosphatidylcholine (PC) in lipopolysaccharide (LPS)-treated MG6 cells.

Fig 7 shows the important intracellular signaling pathways that are triggered by LPS-induced inflammation. First, LPS is recognized by TRL4, which activates the microglia and leads to signaling via the adaptor protein MyD88 [27]. There have been reports of anti-inflammatory substances derived from food and herbal medicine that suppress the expression of TLR4 and MyD88. For example, polymethoxyflavone and *Ganoderma lucidum* extract have been shown to inhibit downstream inflammatory signaling by reducing the expression of TLR4 and MyD88 [28,29]. In our study, egg yolk PC did not affect the TLR4 or MyD88 expression levels in MG6 cells (Fig 6). This finding suggests that egg yolk PC acts downstream of the TLR4/MyD88 signaling pathway. Ishikado et al. reported that soy-derived PC competitively inhibits TLR4, which resulted in the suppression of MCP-1, an inflammatory cytokine mediated by the TLR4-NF-κB signaling pathway, in experiments using human umbilical vein endothelial cells [30]. Interestingly, PC derived from egg yolk did not inhibit MCP-1, which was hypothesized to be due to differences in fatty acid composition. The main fatty acid components of soy PC are palmitic acid, stearic acid, oleic acid, linoleic acid, and α-linolenic acid. In contrast, egg yolk PC contains a higher proportion of saturated fatty acids, such as palmitic acid and stearic acid. In addition to oleic acid and linoleic acid, egg yolk PC includes a wider variety of unsaturated fatty acids, such as arachidonic acid and docosahexaenoic acid (Table 3). Based on the above, the mechanism by which PC affects MG6 may differ depending on its fatty acid composition. DHA is known as a promising molecule that modulates pro-inflammatory microglial activation and cytokine production. Additionally, DHA is known to be more efficiently incorporated into the brain when consumed in the phospholipid form rather than as a triglyceride [31]. Fourrier et al. reported that DHA-containing phospholipids acylated with palmitic acid exhibited high bioavailability in both in vitro and in vivo models and effectively suppressed microglial inflammation [32]. Therefore, the PC used in this study may also serve as an efficient source of DHA.

**Table 3:** Fatty acid composition of phosphatidylcholine.

Fatty acid	% (± SEM)	
C14:0	0.188	± 0.003
C15:0	0.166	± 0.002
C16:0	41.941	± 0.237
C16:1	0.791	± 0.006
C17:0	0.238	± 0.001
C17:1	0.060	± 0.001
C18:0	15.997	± 0.083
C18:1	29.490	± 0.118
C18:2	8.751	± 0.034
C18:3	0.020	± 0.002
C20:0	0.038	± 0.001
C20:2	0.322	± 0.005
C20:4	1.655	± 0.014
C21:0	0.025	± 0.012
C22:4	N.D.	
C22:5	N.D.	
C22:6	0.318	± 0.002

N.D., not detected; SEM, standard error of the mean

Next, we investigated the effects of PC on intracellular signaling molecules. The activated TLR4/MyD88-dependent pathway leads to the induction of proinflammatory cytokines via the activation of the NF-κB and MAPK signaling pathways [33]. NF-κB is a nuclear transcription factor that plays an important role in inflammation [34], and among the NF-κB family, NF-κB p65 is a major subunit in the inflammatory response [35]. Under normal conditions, NF-κB is bound to IκBα in the cytoplasm. However, upon LPS stimulation, IκBα is phosphorylated and degraded, and NF-κB is released and translocated to the nucleus, where it acts as a transcription factor to promote the production of inflammatory cytokines [17,36,37]. In our study, LPS stimulation increased IκBα phosphorylation in the cytoplasm, thereby promoting its degradation and leading to the release of NF-κB p65 from IκBα, which was subsequently translocated into the nucleus (Fig 4). PC treatment suppressed IκBα phosphorylation, thus inhibiting the nuclear translocation of NF-κB p65 (Fig 4). Therefore, one of the mechanisms by which PC inhibits the production of inflammatory cytokines is the suppression of IκBα phosphorylation and the subsequent NF-κB p65 nuclear translocation.

Similar to NF-κB, MAPK is also involved in inflammation-induced signaling cascades, and of the three major families (ERK, p38 MAPK, and JNK), p38 MAPK and JNK have been reported to be responsible for microglial neuron death [21,22]. Upon stimulation, p38 MAPK and JNK phosphorylation occurs, and they are activated. PC treatment suppressed the phosphorylation of JNK and p38 (Fig 3), but not ERK (Supplementary Figure 1). Therefore, it was demonstrated that PC inhibits the downstream MAPK signaling by suppressing JNK and p38, but not ERK. In unstimulated cells, they are present in the cytoplasm as a quiescent form, and activated MAPK promotes the nuclear translocation of the AP-1 complex, which is a dimerized form of c-Jun and c-Fos. The translocation of AP-1 depends on the phosphorylation of p38, ERK, and JNK [38], and the nuclear translocation of the transcription factor AP-1 induces the release of proinflammatory cytokines [18,23,39]. The addition of LPS to MG6 cells caused c-Fos and c-Jun to translocate to the nucleus, but this translocation was suppressed in the PC-treated group (Fig 5). The results indicate that PC inhibits the nuclear translocation of the AP-1 complex, composed of c-Fos and c-Jun, thereby suppressing the production of inflammatory cytokines.

PC, a ubiquitous component of biological membranes, appears to exert its anti-inflammatory effects through two distinct but complementary mechanisms: by serving as a structural substrate for lipid bilayers that maintain mucosal barrier integrity, and by acting as an active modulator of inflammation. PC intake has been shown to protect mucosal surfaces in the stomach and intestine, and delayed-release enteric PC formulations have been used in the treatment of ulcerative colitis to suppress inflammatory activity, leading to both clinical and endoscopic improvement [40–43]. In this context, PC is thought to reinforce the mucosal barrier by supplying essential components for membrane repair and remodeling. In addition to its barrier-supporting role, PC also exhibits direct immunomodulatory activity. For example, egg yolk-derived PC has been reported to ameliorate LPS-induced systemic inflammation and cognitive impairment via modulation of the gut–brain axis [44]. In the present study, we demonstrated that PC suppresses microglial activation, revealing a novel anti-inflammatory mechanism in the central nervous system. This suggests that PC may exert similar effects on peripheral mucosal tissues. In this study, PC treatment suppressed the activation of IκBα, JNK, and p38 in the cytoplasm, rather than influencing TLR4 on the membrane. Previous reports have demonstrated that liposomal PC can permeate into cells, which suggests a high likelihood of cellular uptake [45]. However, it is also possible that PC modulates IκBα, JNK, and p38 phosphorylation through interactions with specific receptors. As this study was conducted in vitro, PC was directly applied to the MG6 cells. The potential for PC to cross the blood-brain barrier and be absorbed into the brain was not examined, and no previous research has investigated this aspect, which is a limitation of the present study. In mouse microglia, β-amyloid has been reported to activate the NLR family pyrin domain containing3 inflammasome via TLR4 [46]. To elucidate the effects of PC in AD and other neurological disorders, it is necessary to conduct in vivo experiments using animal models and to investigate inflammatory pathways involved in pathologies other than those induced by LPS. Furthermore, studies using a Parkinson’s disease mouse model have shown that neuroprotection is associated with alterations in the brain lipid profile, particularly the remodeling of PC, lysophosphatidylcholine, and phosphatidylethanolamine. These findings imply that PC may contribute to the improvement of neurodegenerative conditions such as Parkinson’s disease, not only by serving as a structural lipid essential for membrane homeostasis, but also by exerting neuroprotective effects through lipid-mediated signaling.

In summary, although PC treatment did not affect TLR4/MyD88 activation in LPS-stimulated MG6 microglial cells, it significantly inhibited downstream signaling events, including the phosphorylation of IκBα and JNK/p38 MAPK. Additionally, PC suppressed the nuclear translocation of key transcription factors NF-κB p65 and the AP-1 dimer components c-Fos and c-Jun, resulting in reduced inflammatory cytokine production. While further research using primary microglial cultures and in vivo models is necessary to fully elucidate PC’s therapeutic potential and mechanism of action in neuroinflammatory and neurodegenerative conditions, this study provides the first direct evidence of PC’s anti-inflammatory effects in mouse microglial cells. These findings highlight PC as a promising candidate for modulating microglial activation and neuroinflammation.

## Supporting information

S1 Fig Phosphatidylcholine (PC) does not affect LPS-induced ERK activation in MG6 cells. (PDF)

S1 Raw Images (PDF)

S2 Raw Data (PDF)
