# Supplementary material for: Phosphatidylcholine suppresses inflammatory responses in LPS-stimulated MG6 microglial cells by inhibiting NF-κB/JNK/p38 MAPK signaling
Source: PLoS One. 2025 Jul 28;20(7):e0328206. doi: 10.1371/journal.pone.0328206 (PMC12303320; doi:10.1371/journal.pone.0328206)
Supplement: S1 Fig — (PDF) [file pone.0328206.s001.pdf]

# Supplementary Figure 1. Western Blot Analysis of ERK and Phosphorylated ERK (pERK) Proteins

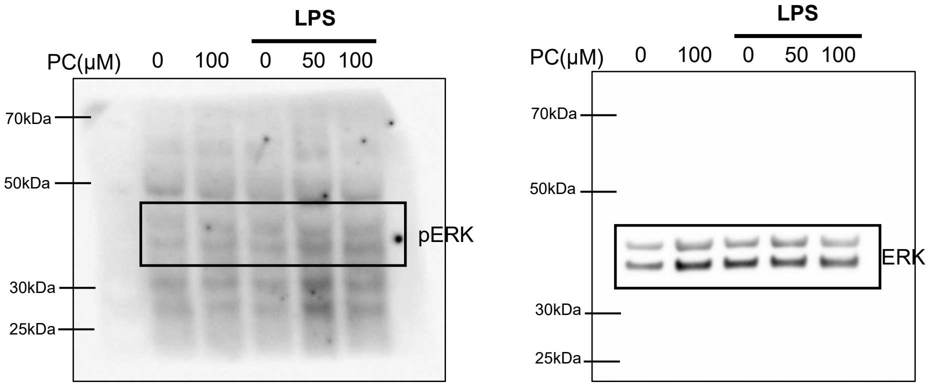


## pERK/ERK

| | | | | | |
|---|---|---|---|---|---|
| | | | LPS (+) | LPS (-) | LPS (+) |
| PC concentration (μM) | 0 | 100 | 0 | 50 | 100 |
| | 0.524661 | 10.83935 | 18.60399 | 27.45711 | 22.58957 |
| | 1.366334 | 17.85653 | 9.890187 | 22.38383 | 31.74843 |
| | 1.113155 | 11.39507 | 15.33519 | 20.94405 | 19.57886 |
