# Supplementary material for: Phosphatidylcholine suppresses inflammatory responses in LPS-stimulated MG6 microglial cells by inhibiting NF-κB/JNK/p38 MAPK signaling
Source: PLoS One. 2025 Jul 28;20(7):e0328206. doi: 10.1371/journal.pone.0328206 (PMC12303320; doi:10.1371/journal.pone.0328206)

Figure 2D full western blot membranes

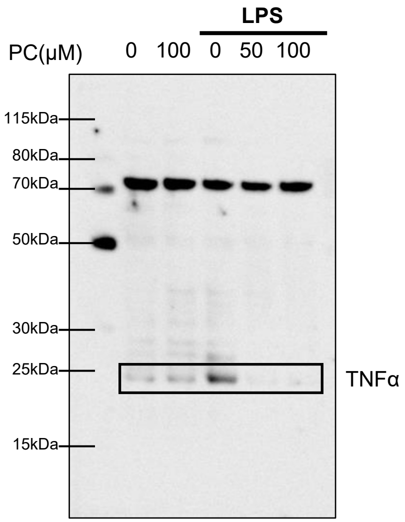

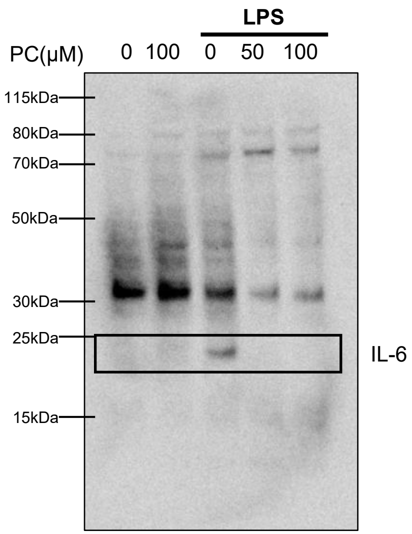

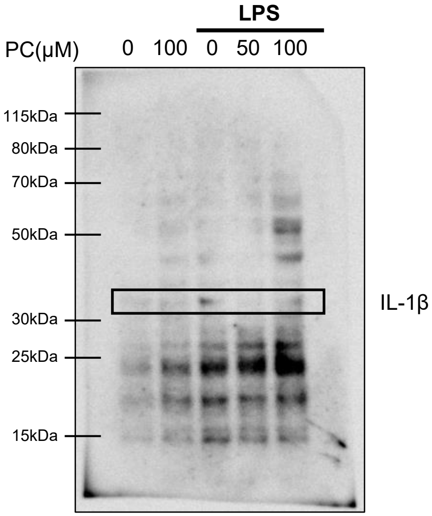

Unprocessed raw image

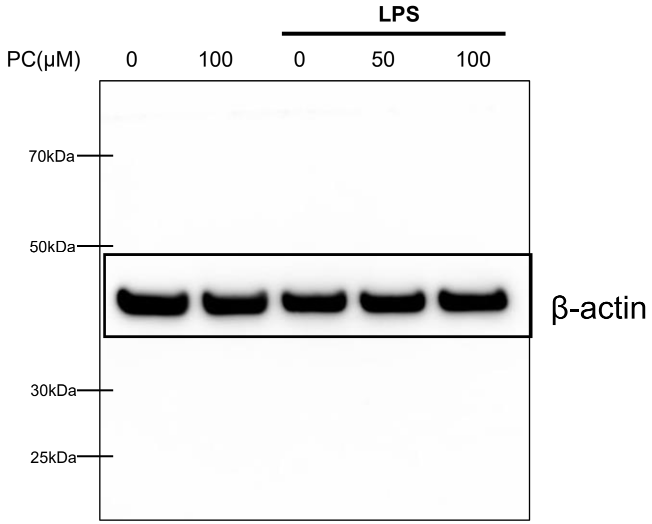

Adjusted for visibility only

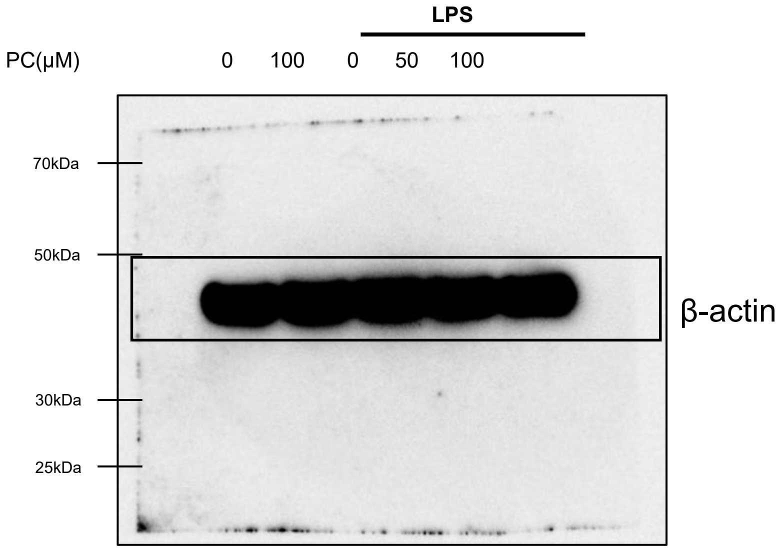

Figure 3A full western blot membranes

Figures 3, 4, 5, and 6 were obtained from the same experiment; therefore, the internal standards β-actin and Lamin B1 used are the same across these figures.

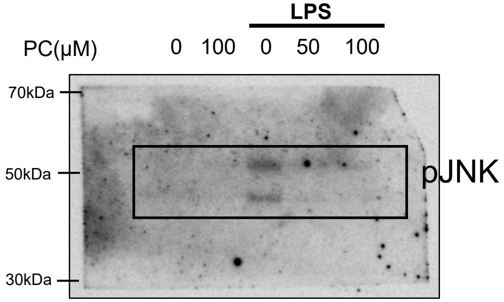

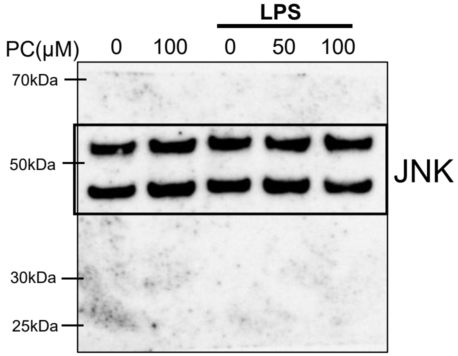

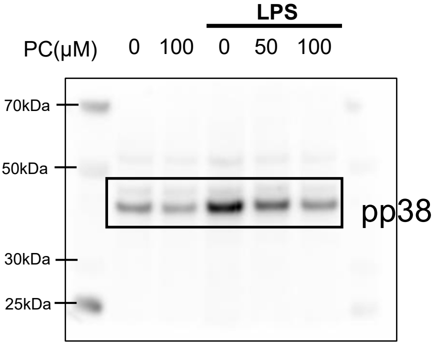

Unprocessed raw image

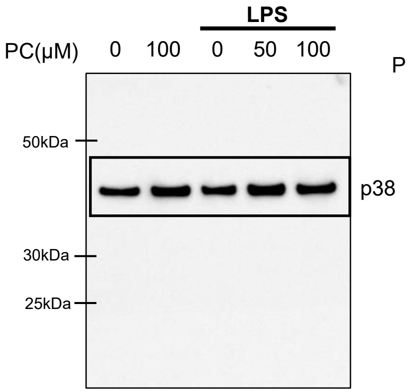

Adjusted for visibility only

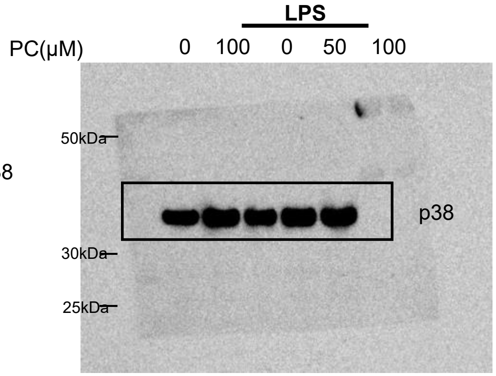

Unprocessed raw image

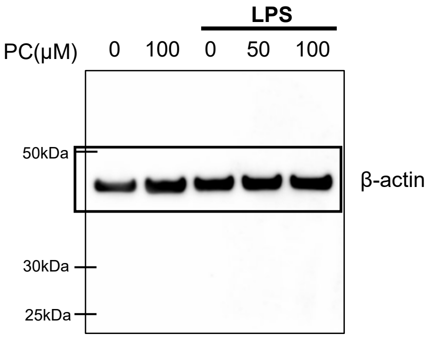

Adjusted for visibility only

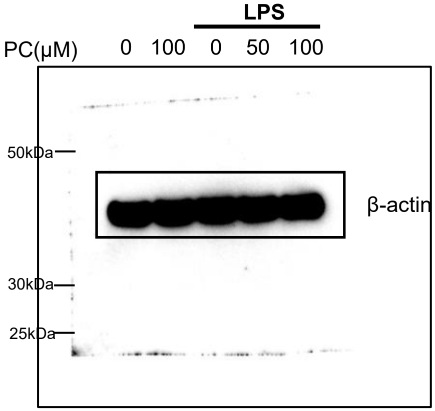

Figure 4A full western blot membranes

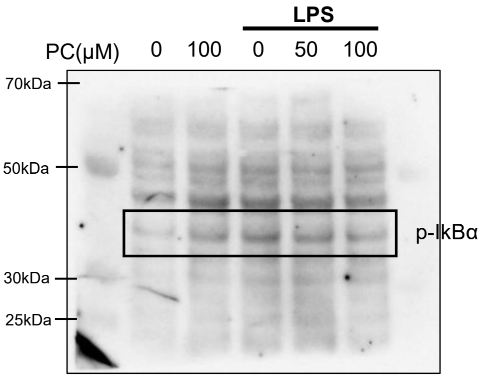

Unprocessed raw image

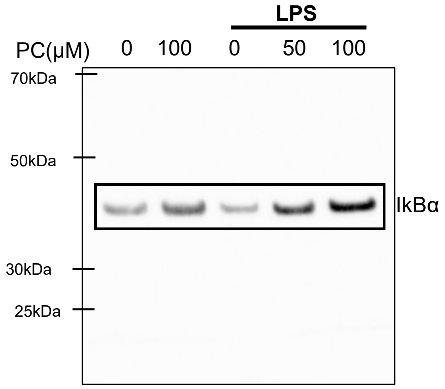

Adjusted for visibility only

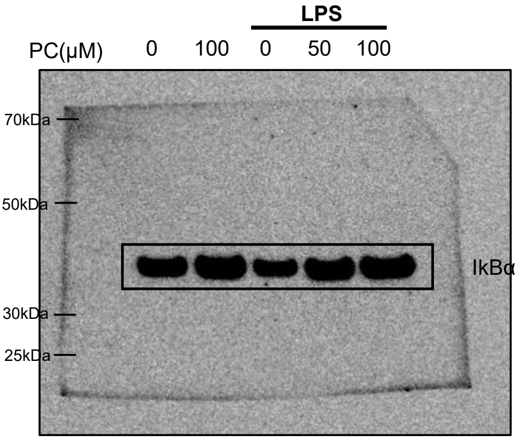

Unprocessed raw image

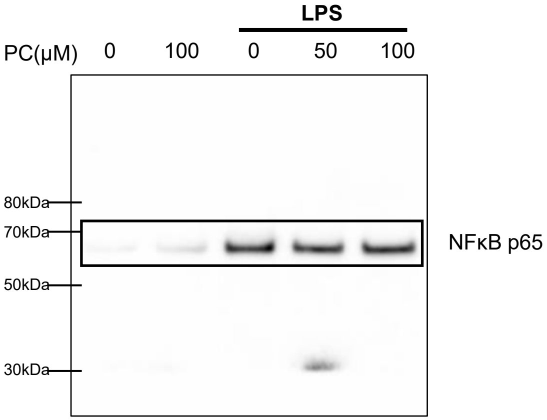

Adjusted for visibility only

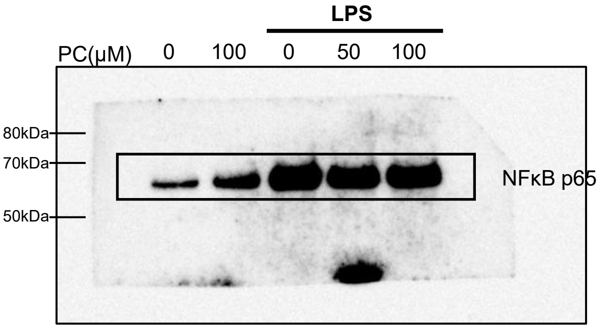

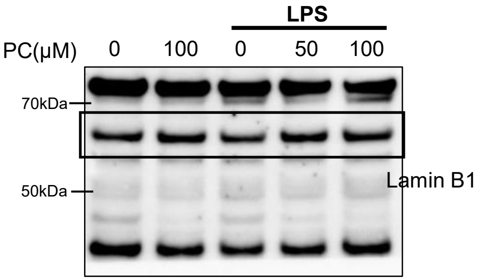

Figure 5A full western blot membranes

Unprocessed raw image

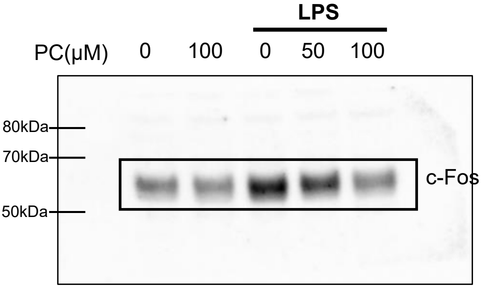

Adjusted for visibility only

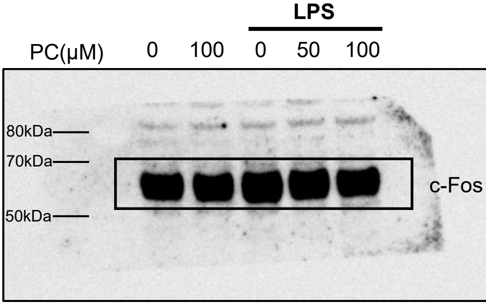

Unprocessed raw image

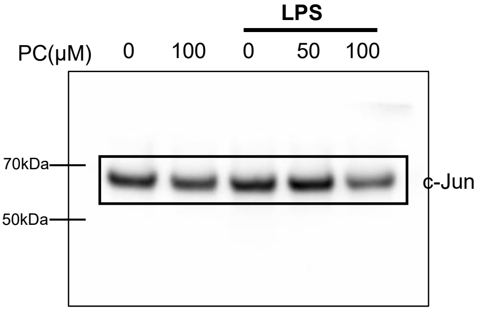

Adjusted for visibility only

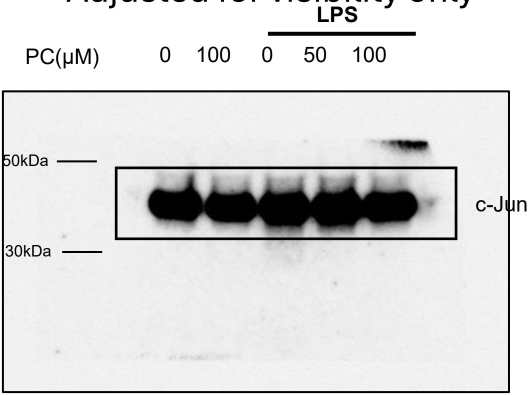

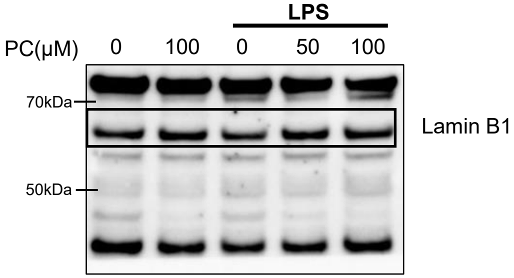

Figure 6A full western blot membranes

Unprocessed raw image

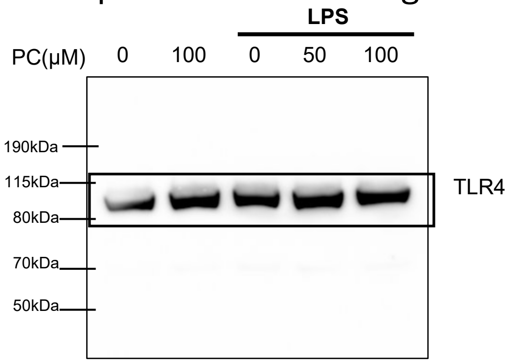

Adjusted for visibility only

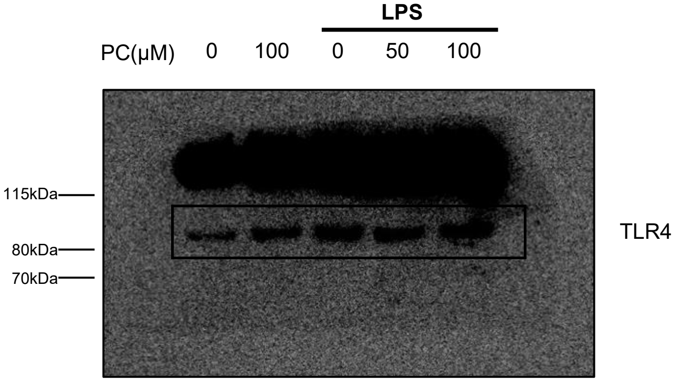

Unprocessed raw image

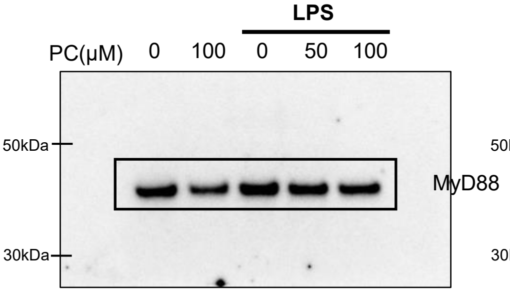

Adjusted for visibility only

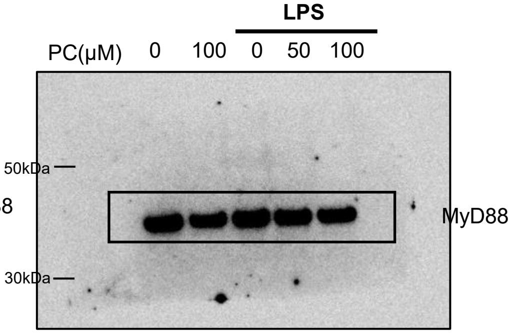

Supplement: S1 Raw Images — (PDF) [file pone.0328206.s002.pdf]
