# Supplementary material for: Phosphatidylcholine suppresses inflammatory responses in LPS-stimulated MG6 microglial cells by inhibiting NF-κB/JNK/p38 MAPK signaling
Source: PLoS One. 2025 Jul 28;20(7):e0328206. doi: 10.1371/journal.pone.0328206 (PMC12303320; doi:10.1371/journal.pone.0328206)
Supplement: S2 Raw Data — (PDF) [file pone.0328206.s003.pdf]

Figure 1 MTT assay results for individual samples

LPS(-) PC100uM(-)	LPS(-) PC5uM(+)	LPS(-) PC20uM(+)	LPS(-) PC30uM(+)	LPS(-) PC50uM(+)	LPS(-) PC100uM(+)
99.90966576	130.532972	113.1887986	119.6928636	85.27551942	104.5167118
111.2917796	130.2619693	114.2728094	119.6928636	112.6467931	105.32972
110.4787715	123.4869015	70.09936766	114.2728094	109.1237579	107.7687444

Figure 2 A-C. individual cytokine mRNA expression data

TNFa/βactin (A)

			LPS (+)	LPS (-)	LPS (+)
PC concentration (μM)	0	100	0	50	100
	0.952638	0.79004131	129.786813	55.3303828	22.3158987
	1.01395948	0.7737825	134.363736	56.1027662	19.6983106
	1.02811383	0.93952275	123.63985	47.176615	17.2676518

IL-1b/βactin (B)

			LPS (+)	LPS (-)	LPS (+)
PC concentration (μM)	0	100	0	50	100
	0.83508792	0.60709744	362.038672	106.891254	32.672388
	0.92018765	0.37113089	369.645874	93.0542411	29.8570557
	1.29235283	0.46976137	326.287521	75.5835303	17.7531116

IL6/βactin (C)

			LPS (+)	LPS (-)	LPS (+)
PC concentration (μM)	0	100	0	50	100
	0.30145196	1.07177346	5042.76752	1722.15586	630.34594
	1.4439292	0.71697762	4039.60916	1499.22375	867.0672
	2.31337637	0.93952275	4482.227	1192.68719	433.5336

Figure 2 E-G individual western blot data.

TNFa/βactin (E)

			LPS (+)	LPS (-)	LPS (+)
PC concentration (μM)	0	100	0	50	100
	1.089554	0.987617	37.42252	1.175838	0.952293
	0.873474	1.021007	77.89718	1.166409	0.831201
	1.03986	1.236659	72.49966	0.994408	0.825698

IL1b/βactin (F)

			LPS (+)	LPS (-)	LPS (+)
PC concentration (μM)	0	100	0	50	100
	11.08884	10.05138	497.0977	11.96699	9.691872
	8.8897	10.3912	719.8078	11.87102	8.459474
	10.58308	12.58598	678.7088	10.12049	8.403464

IL6/βactin (G)

			LPS (+)	LPS (-)	LPS (+)
PC concentration (μM)	0	100	0	50	100
	1.090676	0.988634	434.627	1.177049	0.953273
	0.874373	1.022058	708.5056	1.16761	0.832057
	1.04093	1.237932	589.7104	0.995431	0.826548

Figure 3 B-C individual western blot data.

pJNK/JNK

			LPS (+)	LPS (-)	LPS (+)
PC concentration (μM)	0	100	0	50	100
	0.08414	0.898362	6.897536	2.775217	0.554572
	1.018241	0.849788	5.183336	0.907457	1.465187
	1.879563	0.715915	4.766195	1.096256	0.663796

pp38/p38

			LPS (+)	LPS (-)	LPS (+)
PC concentration (μM)	0	100	0	50	100
	0.854979	1.015706	3.140024	2.39098	1.258939
	0.999604	1.388198	2.209176	2.01841	1.769334
	1.145828	1.017118	3.576281	1.855168	1.435729

Figure 4 B-C individual western blot data.

pIkB/IkB

			LPS (+)	LPS (-)	LPS (+)
PC concentration (μM)	0	100	0	50	100
	0.509121	0.463383	30.41305	17.70533	1.049531
	1.88053	1.864652	72.84854	11.18901	0.693321
	0.611408	2.640811	69.19226	7.434168	2.975611

NFkB p65(N)/LaminB1

			LPS (+)	LPS (-)	LPS (+)
PC concentration (μM)	0	100	0	50	100
	1.152255	0.521821	2.199929	1.082082	0.86028
	1.33413	0.432848	3.068895	0.80756	1.642482
	0.847667	1.107127	2.050498	0.682376	1.154389

Figure 5 B-C individual western blot data.

c-fos/LaminB1

			LPS (+)	LPS (-)	LPS (+)
PC concentration (μM)	0	100	0	50	100
	1.307618	0.464412	2.106245	1.603759	1.082969
	0.89427	0.531786	3.048867	1.53169	0.755382
	0.798551	1.144357	3.579961	1.614815	0.713679

c-jun/LaminB1

			LPS (+)	LPS (-)	LPS (+)
PC concentration (μM)	0	100	0	50	100
	0.962999	0.773103	3.656249	2.832321	2.036258
	1.087959	0.944879	2.47476	2.331077	1.639463
	0.948732	1.297909	2.646844	2.983535	1.396144

Figure 6 B-C individual western blot data.

MyD/βactin

			LPS (+)	LPS (-)	LPS (+)
PC concentration (μM)	0	100	0	50	100
	1.068996	1.09044	1.653567	0.639401	1.677738
	1.045546	1.017769	1.460058	1.240078	1.494572
	0.903882	1.204025	1.695364	1.300415	1.275887

pERK/ERK

			LPS (+)	LPS (-)	LPS (+)
PC concentration (μM)	0	100	0	50	100
	0.524661	10.83935	18.60399	27.45711	22.58957
	1.366334	17.85653	9.890187	22.38383	31.74843
	1.113155	11.39507	15.33519	20.94405	19.57886

Table 3. Fatty acid composition of phosphatidylcholine

Fatty acid (%)			
C14:0	0.190974	0.18902	0.184373
C15:0	0.16637	0.166762	0.163531
C16:0	42.12814	42.01969	41.67518
C16:1	0.79574	0.793429	0.78435
C17:0	0.237415	0.237381	0.239751
C17:1	0.058714	0.061455	0.061124
C18:0	15.93055	15.97094	16.0896
C18:1	29.39653	29.45149	29.62249
C18:2	8.725742	8.736798	8.788984
C18:3	0.019542	0.018934	0.021842
C20:0	0.036817	0.037521	0.038824
C20:2	0.319203	0.3191	0.326948
C20:4	1.645102	1.648516	1.670365
C21:0	0.032756	0.031219	0.01175
C22:4	N.D.	N.D.	N.D.
C22:5	N.D.	N.D.	N.D.
C22:6	0.316407	0.317742	0.320888
